# A Novel Candidate Gene *MACF1* is Associated with Autosomal Dominant Non-syndromic Hearing Loss in an Iranian Family

**DOI:** 10.34172/aim.31746

**Published:** 2025-01-01

**Authors:** Niloofar Bazazzadegan, Mojgan Babanejad, Susan Banihashemi, Sanaz Arzhangi, Kimia Kahrizi, Kevin TA Booth, Hossein Najmabadi

**Affiliations:** ^1^Genetics Research Center, University of Social Welfare and Rehabilitation Sciences, Tehran, Iran; ^2^Department of Medical and Molecular Genetics, Indiana School of Medicine, Indianapolis, IN, USA; ^3^Department of Otolaryngology-Head and Neck Surgery, Indiana School of Medicine, Indianapolis, IN, USA

**Keywords:** Autosomal dominant non-syndromic hearing loss, Iran, MACF1, Novel gene

## Abstract

Cytoskeletal dynamics, the interplay of actin, microtubules, and septins, is a highly coordinated and tightly regulated process. Defects in the proteins involved can result in a wide range of cellular consequences. Hearing loss is the most common sensory defect and exhibits extraordinary genetic and phenotypic heterogeneity. Currently, there are more than 170 genes casually linked to non-syndromic hearing loss (NSHL), of which more than 60 are associated with autosomal dominant inheritance. Here, we add to this growing number by implicating *MACF1* (OMIM # 608271), as a novel candidate gene for autosomal dominant non-syndromic hearing loss (ADNSHL). MACF1’s cytoskeleton integrator function and hair cell expression pattern lead one to believe that it is a necessary protein for hair cells. Many protein domains in MACF1 allow for dynamic interaction with the cytoskeleton. A large Iranian family segregating progressive ADNSHL was recruited for this study. The proband had bilateral mild-moderate sensorineural hearing loss and was negative for *GJB2* mutations. After applying exome sequencing on the proband, a missense mutation c.1378C>T (p.His460Tyr) was found in *MACF1* and co-segregated with the hearing loss in the extended family. We speculated that *MACF1* mutations probably cause non-syndromic hearing loss inherited in an autosomal dominant manner. The potential functional impact of the identified variant will be investigated through further analysis.

## Introduction

 Congenital sensorineural hearing loss (HL) affects ~1 of every 1000 live births.^1^ This rises to 2.8 per 1000 in school-age children and to 3.5 per 1000 adolescents.^2^ In developed countries, it is estimated that ~80% of HL has a genetic etiology. After clinical evaluation, comprehensive genetic testing is the next best test to determine clinical actions and interventions, and to provide a definitive diagnosis. This allows for identification of the underlying genetic cause, facilitating tailored management strategies, genetic counseling, and prognosis determination.

 Genetic HL displays a vast genetic allelic and phenotypic spectrum.^3^ Currently, comprehensive genetic testing for HL returns positive results from a 35%‒50% diagnostic rate, depending on several variables such as: phenotype, onset, inheritance pattern, and ethnicity. This diagnostic rate illustrates the complexity of providing a genetic diagnosis, and implicates the contributions of novel genes to genetic HL that have yet to be identified.^4^

 Currently, there are more than 170 genes casually linked to non-syndromic hearing loss (NSHL), of which more than 60 are associated with autosomal dominant inheritance (AD) (org https://hereditaryhearingloss.org/). Here, we add to this list by implicating *MACF1* as a possible novel gene for postlingual progressive ADNSHL.

## Case Report

**Figure 1:**
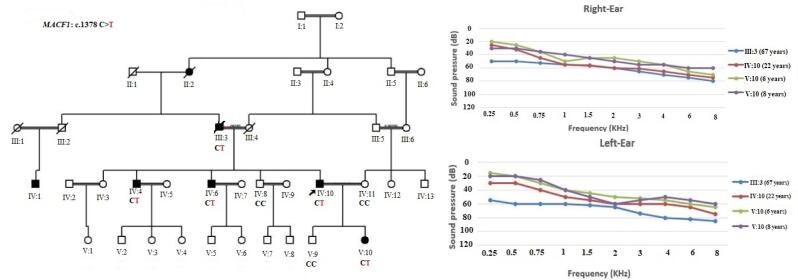
Family Pedigree with Genotype of Affected and Normal Individuals (Affected individual has been shown with C/T genotype and normal individual with C/C). Pure tone audiometry for right and left ears of proband, proband’s father and daughter

**Figure 2:**
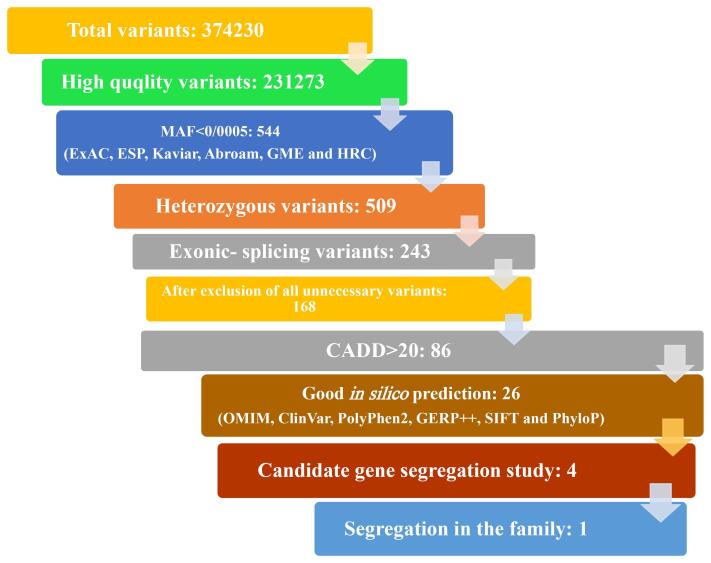
Variant Filtering and Prioritization Based on *In-Silico* Predictions (PolyPhen2, GERP++, SIFT, and PhyloP)

 The proband presented to the genetics clinic at University of Social Welfare and Rehabilitation Sciences. The proband has an extensive family history of HL (Figure 1), accompanied by no other phenotypic manifestations. After obtaining informed consent, whole blood samples were collected from participating members and genomic DNA was extracted (Figure 1). Affected members of the family underwent clinical re-evaluation to rule out potential missed syndromic forms of HL. Pure tone audiometry was performed on affected individuals and revealed a mild sloping to severe HL. The HL is described as postlingual, and progressive. Individual III.3 showed a more severe HL in the low frequencies and may represent some progression of the low frequencies with age. Initially, the proband underwent *GJB2* testing, which revealed no causal mutations. Subsequently, the proband underwent exome sequencing (ES) to determine the genetic cause of HL segregating in the family. After read mapping and quality filtering, the exome data was analyzed using a tiered approach. First, variants in genes causally linked to HL were reviewed and no plausible variant was identified, for either AD or autosomal recessive NSHL. Next, a broader search was employed. Variants were filtered based on minor allele frequency [ESP6500 (http://evs.gs.washington.edu/EVS), ExAC (https://exac.broadinstitute.org/), Iranome (http://www.iranome.ir/), and gnomAD (http://gnomad.broadinstitute.org/)], inheritance pattern and predicted variant consequence. Next, variants were prioritize based on *in-silico* predictions [PolyPhen2 (http://genetics.bwh.harvard.edu/pph2/), GERP + + , SIFT (http://sift-dna.org) and PhyloP) and classifications in public databases [OMIM (https://www.omim.org/) and ClinVar (https://www.ncbi.nlm.nih.gov/clinvar/)] (Figure 2) (Supplementary file 1, Table S1).

 After applying the filtering above, four variants were further prioritized for segregation within the extended family. Of these, only the heterozygote missense variant (GRCh37/hg19ch:1, 39751285, NM_012090.5, c.1378C > T; p.His460Tyr) in *MACF1* co-segregated with the HL phenotype (Table 1; Figure S1). Forward primer: 5’-AGACTTCTTGGCTCCCTCTG-3’ and reverse primer: 5’-GAGTCCCTTGTTCCTCACCT-3’ were used for Sanger sequencing of the detected variant. This variant has a minor allele frequency (MAF) 0.000004 (GnomAD_exomes) and 0.000008 (ExAC). It has not been reported in Iranome which is a domestic population database.

**Table 1:** Databases and *In Silico* Algorithms for *MACF1* and Three Other Candidate Variants Undergoing Segregation Analysis

**Gene**	**Variant**	**CADD**	**GERP**	**SIFT**	**Phylop**	**Polyphen**	**Franklin**	**InterVar**	**Clinvar**	**MIM#**	**Segregation**
*MACF1*	c.1378C > T	23.3	5.93	0.003	3.72	—	VUS	Likely pathogenic	—	608271	Yes
*DNAH14*	c.1990-1991insTT	—	—	—	—	—	Likely pathogenic	—	—	603341	No
*USP6*	c.G2763A	38	2.91	—	9.74	—	VUS	VUS	—	604334	No
*MUC16*	c.40674-40677del	—	—	—	—	—	VUS	—	—	606154	No

 The histidine in position 460 was changed with tyrosine which is an aromatic amino acid. According to HOPE results (https://www3.cmbi.umcn.nl/hope/method/), the wild-type and mutant amino acids differ in size. The mutant residue is bigger which might lead to local misfolding. The hydrophobicity of the wild-type and mutant residue differs. The mutation introduces a more hydrophobic residue at this position. This can result in loss of hydrogen bonds and/or disturb correct folding.

## Discussion

On human Chr 1p32, the *MACF1* gene is located near the DFNA2 dominant HL locus. It has been demonstrated that HL in certain DFNA2 families is caused by mutations in *KCNQ4*, which are centromeric to *MACF1.*^5-7^ No plausible variants in *KCNQ4* were detected in the proband based on ES analysis. The microtubule and actin crosslinking factor 1 (*MACF1*) gene encodes Actin Crosslinking Family Protein 7 (MACF1), a massive (~500 kDa) cytoskeletal crosslinking protein that interacts with F-actin and microtubules to shape cell morphology.^6^ Metazoan tissues have widespread expression of MACF1, indicating a notable degree of evolutionary conservation.^8^ MACF1’s cytoskeleton integrator function and hair cell expression pattern lead one to believe that it is a necessary protein for hair cells. The developmentally significant nature of microtubule and actin crosslinking factor 1 genes is established by the embryonic lethality of a null mutation in the mouse ortholog.^9^ Moreover, a mutation in zebrafish causes abnormalities in the oocyte and egg’s animal-vegetal polarity.^10^ Many protein domains in MACF1 allow for dynamic interaction with the cytoskeleton. Direct contact between calponin homology 1 and 2 domains and F-actin is facilitated towards the N terminus. The GSR (Gly-Ser-Arg)-repeat domain, which also interacts with microtubules, the EF hand domains, which bind calcium, and the GAS2-related protein domain, which binds with microtubules and helps microtubule stabilization, are located close to the C-terminus.^9^

Interestingly, although highly expressed, MACF1 is not required for normal hair cell development and maturation, and conditional knockout cKO mice for MACF1 have normal hearing at P30.^11^ We speculate two possibilities to explain the phenotype seen in humans but not the cKO mouse. First, it is possible that MACF1 is dispensable for normal hair cell development and maturation but might play a more important role throughout the life of the hair cell, and examination of its absences at P30 might not capture its true biological role. Second, we identified a missense variant which is hypothesized to act as a gain-of-function or dominant negative and the cKO mouse is not a good model to recapitulate this effect. It is noteworthy that *MACF1* lies ~1.2 Mb downstream of *KCNQ4­, *andno plausible variants were detected in the exonic regions of *KNCQ4*.

Currently, variants in *MACF1* are causally linked to Lissencephaly 9 with complex brainstem malformation (LIS9). LIS9 is an autosomal dominant form of lissencephaly, which is a disorder that affects the development of the brain’s cortex. This disorder is characterized by the absence or thickening of the normal six-layered cortex, leading to disorganization. Clinically, LIS9 is associated with global developmental delay that is noticeable from infancy. Individuals with LIS9 also experience impaired intellectual development, often resulting in poor or absent speech. Additionally, abnormal or involuntary movements may be present. Brain imaging reveals malformation of the brainstem, as well as the presence of pachygyria and lissencephaly (MIM:618325). As it was obvious in our family, no syndrome was detected and only HL was found in the affected individuals.

## Conclusion

 In conclusion, we report a novel candidate gene (*MACF1*) for autosomal dominant non-syndromic hearing loss (ADNSHL). Functional studies are needed to understand the impact of the p.His460Tyr variant on MACF1 function and how this variant results in HL. Additionally, identifying more families segregating NSHL and variants in *MACF1* will help provide a better understanding of MACF1 phenotypic spectrum. Finally, we have expended the phenotypic spectrum of MACF1 to include ADNSHL.

## Supplementary Files

Supplementary file 1 contains Figure S1 and Table S1.
