# Supplementary material for: A Novel Candidate Gene MACF1 is Associated with Autosomal Dominant Non-syndromic Hearing Loss in an Iranian Family
Source: Arch Iran Med. 2025 Jan 1;28(1):63–6. doi: 10.34172/aim.31746 (PMC11862395; doi:10.34172/aim.31746)
Supplement: Supplementary file 1 — contains Figure S1 and Table S1. [file aim-28-63-s001.pdf]

Arch Iran Med. January 2025;28(1):63-S1

10.34172/aim.31746

http://journalaim.com

Supplementary file 1

Figure S1: Electropherogram of affected and unaffected individuals of family L-8500213 for segregation analysis of *MACF1*, *DNAH14*, *MUC16* and *USP6* genes. In *MACF1*, all affected persons are heterozygote for the c.C1378T and all normal individuals are homozygote for the wild allele, but for three other genes segregation in the family was not confirmed. In *MUC16*, variant was not confirmed in proband too.

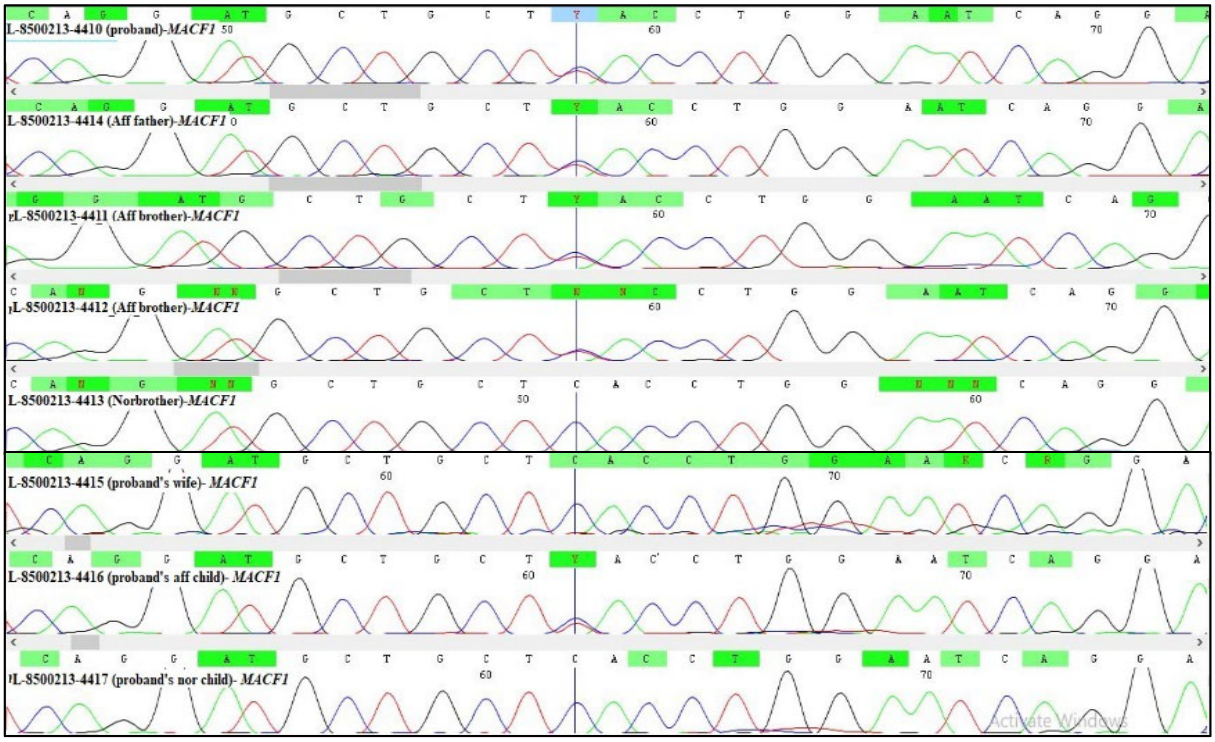

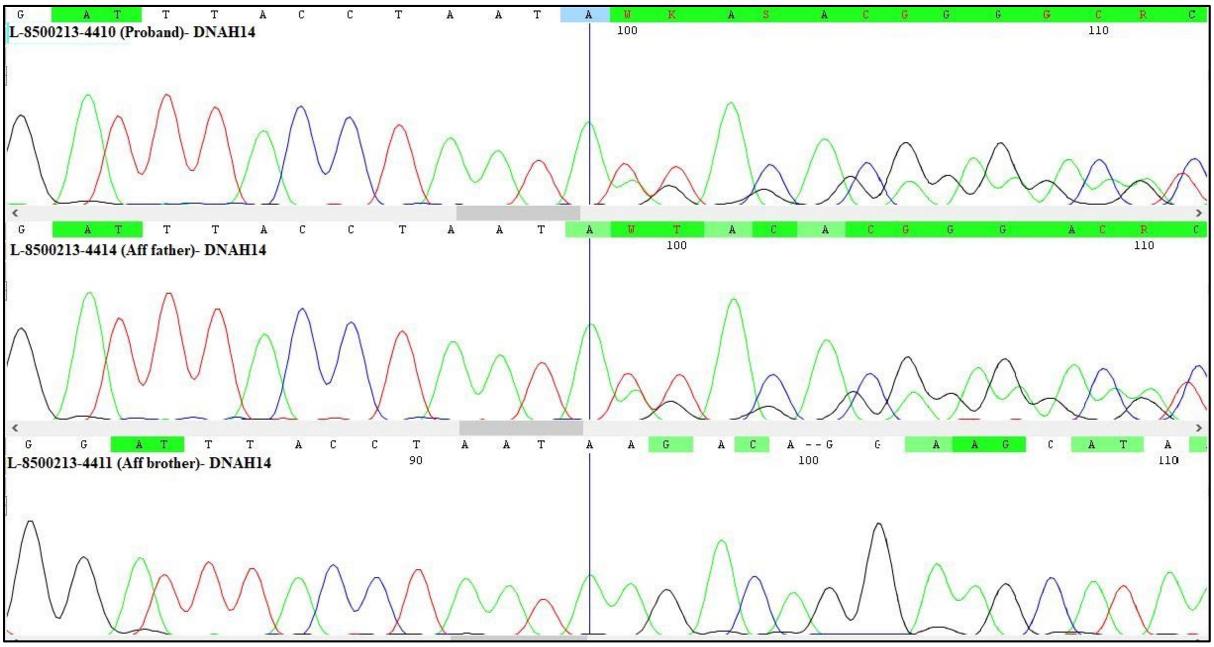

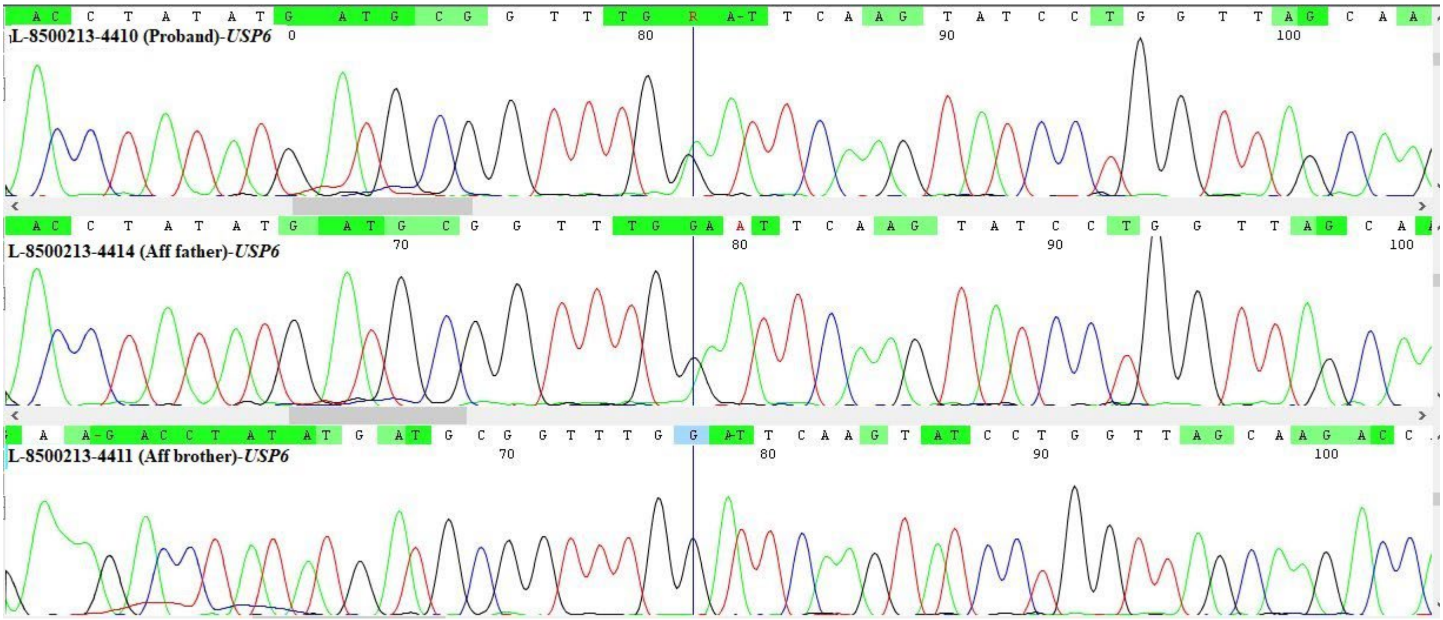
L-8500213-4410 (Proband)-USP6
L-8500213-4414 (Aff father)-USP6
L-8500213-4411 (Aff brother)-USP6

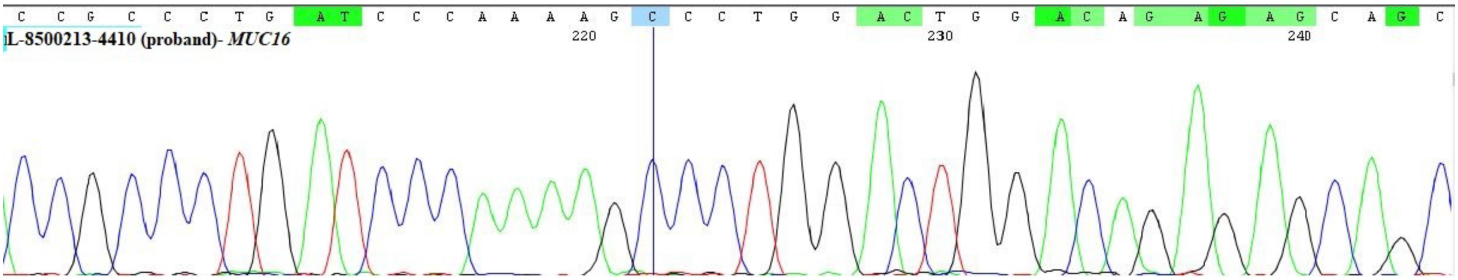
L-8500213-4410 (proband)- MUC16

Table S1: All of candidate variants and ones which have been performed segregation analysis

Gene	Chromosome position	Variant	Segregation
DNAH14	Chr1: 225237989	c.1990-1991insTT	No
USP6	Chr17: 5058836	c.G2763A	No
MUC16	Chr19: 8999498	c.40674-40677del	No
MACF1	Chr1: 39751285	c.C1378T	Yes
NLRC4	Chr2: 32476071	c.C862T	–
NCOA5	Chr20: 44699165	c.G49A	–
SLC25A5	ChrX: 118604428	c.T691C	–
MYH6	Chr14: 23858093	c.C4150T	–
INPP1	Chr2: 191235908	c.T980C	–
ANK3	Chr10: 61834991	c.C5648T	–
INTS1	Chr10: 1536799	c.C1577T	–
PABPC3	Chr13: 25671168	c.C832T	–
GTF3C2	Chr2: 27559149	c.A1271G	–
CRB2	Chr9: 126125419	c.C370T	–
WASHC2A	Chr10: 51829421	c.A241G	–
ITGA7	Chr12: 56087806	c.C1190T	–
SLC25A5	ChrX: 118604444	c.G707C	–

AGAP2	Chr12: 58125741	c.G1804A	-
CCDC47	Chr17: 61829377	c.C1294T	-
NDUFS2	Chr1: 161182257	c.G1103A	-
ALG1L	Chr3: 125648332	c.C367T	-
ADAMTS2	Chr5: 178772229	c.99_101del	-
PABPC3	Chr13: 25671150	c.814_816del	-
PABPC3	Chr13: 25671155	c.819_827del	-
LVRN	Chr5: 115351067	c.2569_2570insTCACAGATGCTGGCTA	-
NBPF9	Chr1: 145368473	unknown	-
